# Editorial: Endocrinological sequelae of hematopoietic stem cell transplantation

**DOI:** 10.3389/fendo.2023.1151213

**Published:** 2023-02-14

**Authors:** Valentina Giudice, Carmelo Gurnari, Simona Pagliuca, Maria Antonietta Castaldi, Carmine Selleri

**Affiliations:** ^1^ Hematology and Transplant Center, University Hospital “San Giovanni di Dio e Ruggi d’Aragona”, Salerno, Italy; ^2^ Department of Medicine and Surgery, University of Salerno, Baronissi, Italy; ^3^ Department of Biomedicine and Prevention, University of Rome Tor Vergata, Rome, Italy; ^4^ Translational Hematology and Oncology Research, Department of Cleveland Clinic, Cleveland Clinic, Cleveland, OH, United States; ^5^ Service d’hématologie, Hôpital Brabois, Nancy Clinic, 7365 IMoPa, Biopôle de l’Université de Lorraine, Vandoeuvre les Nancy, France; ^6^ Obstetrics and Gynecology Unit, University Hospital “San Giovanni di Dio e Ruggi d’Aragona”, Salerno, Italy

**Keywords:** endocrinological sequalae, hematopoietic stem cell transplantation, long-term follow-up, cancer survivorship, personalized medicine

## 1 Introduction

**Figure 1:**
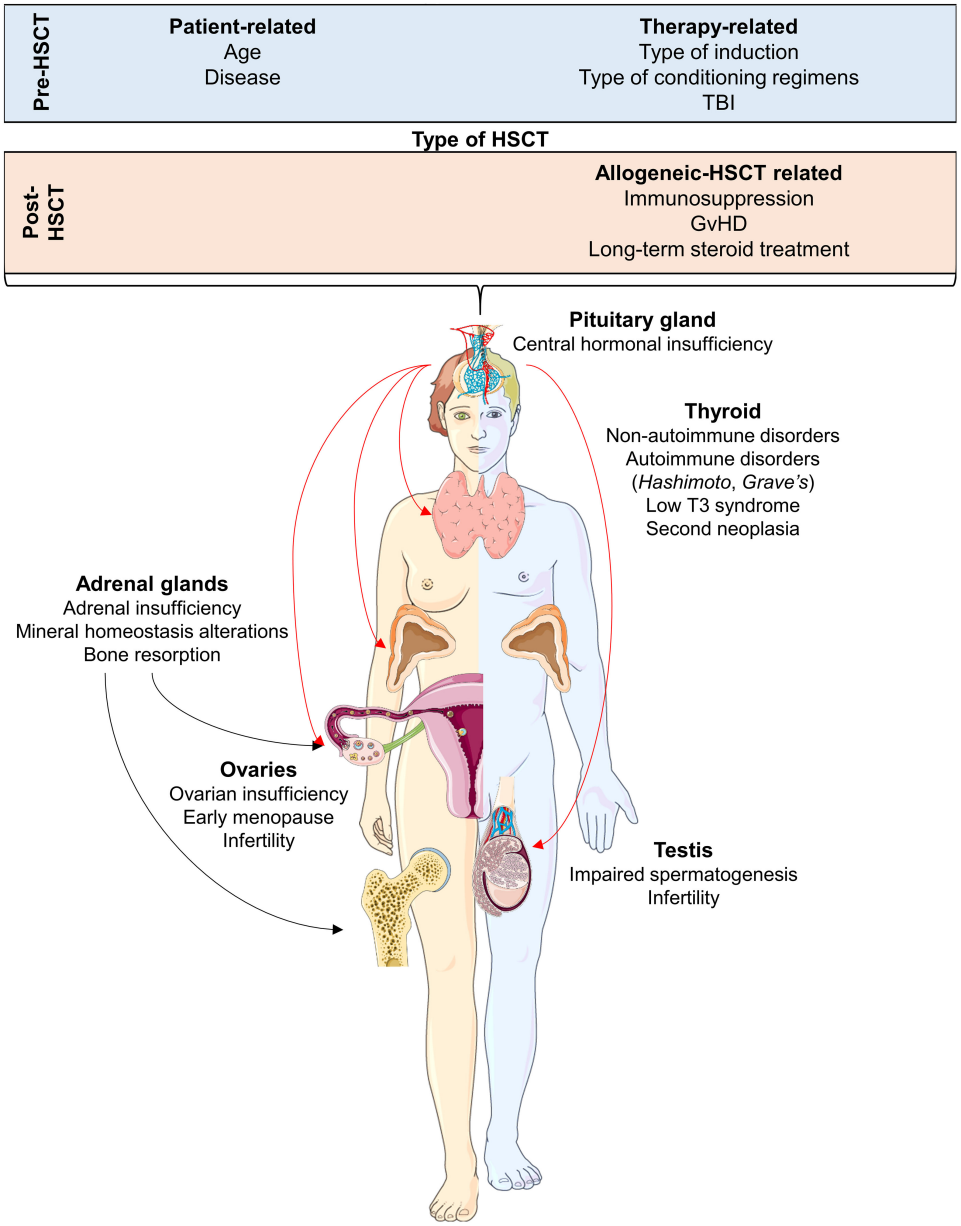
Endocrine complications after hematopoietic stem cell transplantation (HSCT) for each organ and tissue affected. Pre- and post-transplant conditions that are considered as risk factors of endocrine sequelae are also displayed. Abbreviations. TBI, total body irradiation; GvHD, Graft versus Host DIsease. Parts of the figure were drawn by using pictures from Servier Medical Art. Servier Medical Art by Servier is licensed under a Creative Commons Attribution 3.0 Unported License (https://creativecommons.org/licenses/by/3.0/).

Autologous (auto-) and allogeneic (allo-) hematopoietic stem cell transplantation (HSCT) remains the only curative therapeutic strategy for several benign and malignant hematological disorders, such as inherited bone marrow failure syndromes and acute leukemias (1). Transplant-related complications, including severe myelosuppression, graft failure, and graft versus host disease (GvHD), are the major causes of poor outcomes post-HSCT and reduced quality of life. Among other challenges, GvHD-related symptoms and disorders (such as bronchiolitis obliterans organizing pneumonia with decreased pulmonary function or sclerodermatous-like chronic GvHD) and secondary cancers significantly impact post-HSCT outcomes (2). Moreover, long-term survivors may experience early and late endocrine disorders, which may occur as different clinical manifestations of the GvHD-related immune dysregulation, or may independently arise as late effects of pre-transplant induction and/or conditioning regimens (3). Underlying hematological condition, age at transplant, pre-HSCT therapies and cumulative doses of anticancer drugs, and conditioning regimens with or without total body irradiation (TBI) are the main risk factors for the development of endocrine complications after transplant (4). Iatrogenic immunosuppression induced by conditioning regimens and GvHD prophylaxis or therapies with prolonged administration of immunosuppressive drugs and/or steroids also deeply alter the functions of the endocrine system, further contributing to the development of post-transplant endocrine dysregulation and disorders. These endocrine sequelae can affect all endocrine tissues, including the male and female gonads, hypothalamus, pituitary gland, thyroid, pancreas, adrenal glands, and bone metabolism (**Figure 1**) (3).

In this collection of Frontiers in Endocrinology articles, we have collated original research articles and reviews that offer an overview of endocrine complications after HSCT; this may help to provide a better understanding of the long-term clinical outcomes of transplant survivors. Furthermore, the collected articles provide an update on treatment strategies to improve the clinical management of these complications.

In their review, Miglietta et al. summarize the biological mechanisms underlying post-HSCT impairment of calcium-tropic organs involved in mineral homeostasis, such as the kidneys, bones, and intestines, which leads to bone resorption and early osteoporosis. The latter is a frequent post-HSCT complication caused by steroid-based pre-transplant regimens and immunosuppressive agents for GvHD prophylaxis (5). Moreover, pharmacological and clinical management strategies are presented for post-transplant altered mineral homeostasis, renal dysfunction, and bone loss, including novel targeted therapies, such as romosozumab.

The review by Bai et al. systematically investigates the role of osteopontin (a multifunctional phosphoprotein secreted by many cell types, such as osteoclasts and chondrocytes, that are present in the extracellular matrix of mineralized tissues) in physiological and pathological conditions, especially osteoarthritis and osteoporosis. This review elucidates the involvement of osteopontin in various biological mechanisms, collating evidence from all significant studies, and also highlights possible pharmacological interventions for treatment of osteoporosis involving targeting of osteopontin-related signaling pathways.

The study by Immediata et al. explores the reasons why female patients affected by cancer who are undergoing high-dose chemotherapy, including conditioning regimens, frequently agree to oocyte cryopreservation but rarely use the cryopreserved specimens for fertility procedures. Notably, recurrent malignancy or ongoing anticancer therapies are only marginal reasons, while Immediata et al. highlight a clearer role for gender biases relating to the ideas of motherhood and pregnancy.

The review by Cattoni et al. tackles one of the most common form of endocrinological sequelae post-HSCT, namely thyroid function disorders and secondary thyroid cancers (6). With long-term rates of up to 70%, thyroid disorders require lifelong monitoring and span from hypo/hyperthyroidism to the occurrence of autoimmunity, nodules, and secondary cancers. In their article, Cattoni et al. provide an overview of the various risk factors (TBI, young age at HSCT) and propose a rational approach to this specific aspect of cancer survivorship.

In a meta-analysis, Lu et al. explore global research trends in steroid-induced osteonecrosis of the femoral head (SONFH), another classic post-HSCT complication that is typically related to the use of steroids to prevent GvHD in the early post-transplant period. The authors present an overview of the relevant global research trends and specific avenues of investigation, identifying genomics and molecular cell biology as the current research frontiers for SONFH.

Finally, Gavin et al. use *in vivo* mouse models to investigate the impact of hematopoietic stem cell-derived adipocytes (HSCDAs), a distinctive white fat subpopulation of adipocytes deriving directly from hematopoietic progenitors. While they did not find that depletion of HSCDAs significantly impacted adipose depot weights, the number of small adipocytes and conventional adipocyte progenitors did increase. These changes were accompanied by increased insulin resistance, with higher leptin levels and a decrease in physical activity occurring in parallel.

## Author contributions

All authors listed have made a substantial, direct, and intellectual contribution to the work, and approved it for publication.
